# Left Ventricular Diastolic Response to Isometric Handgrip Exercise in Physically Active and Sedentary Individuals

**DOI:** 10.3390/jcdd9110389

**Published:** 2022-11-11

**Authors:** Dimitrios Rovithis, Maria Anifanti, Nikolaos Koutlianos, Andriana Teloudi, Evangelia Kouidi, Asterios Deligiannis

**Affiliations:** Laboratory of Sports Medicine, School of Physical Education and Sport Science, Aristotle University of Thessaloniki (AUTh), 57001 Thessaloniki, Greece

**Keywords:** diastolic LV function, isometric handgrip exercise, lusitropic cardiac function, high physical activity levels, stress echocardiography

## Abstract

Aims: This study aims to investigate the diastolic left ventricular (LV) response to isometric handgrip exercise among healthy middle-aged men with high physical activity levels, versus matched sedentary individuals. Methods: Two groups of 10 men aged 41–51 years were studied. Men in the first group had high weekly self-reported physical activity levels (>3000 METs × min/week). In comparison, men in the second group reported low physical activity levels (<300 METs × min/week). An isometric handgrip exercise (IHE) stress echocardiography test was performed in all of them. Results: Both groups showed a similar and statistically significant increase in heart rate, systolic, diastolic, and mean arterial pressure following IHE. The group of active men under study did not show a statistically significant change in the ratio of early diastolic mitral valve inflow velocity to early diastolic lateral wall tissue velocity (E/e’ ratio) in response to IHE. Conversely, the inactive participants’ E/e’ ratio was higher at peak activity in the isometric handgrip exercise. Conclusions: Apparently, healthy middle-aged men with high levels of physical activity seem to have an improved lusitropic cardiac function compared to men with low levels of physical activity, as observed by the different diastolic LV responses induced by isometric handgrip exercise.

## 1. Introduction

The diastolic function of the left ventricle (LV) is a crucial component of its overall cardiac output. Its perfect functioning plays an essential role in total physical performance. The correct assessment of LV diastolic function is challenging in clinical practice due to multiple intrinsic and extrinsic factors mediating LV filling [1]. Exercise training was found to enhance LV compliance and myocardial relaxation and also reverse diastolic dysfunction in some abnormal conditions [2,3]. On the contrary, physically inactive patients were likelier to have diastolic dysfunction [4]. Studies focusing on the diastolic function in athletes as a cardiac adaptation to long-term exercise training are limited and are often inconclusive [5]. Several papers documented enhanced LV diastolic function in elite athletes, using Doppler, tissue-Doppler, strain, and other relevant methods; some authors have observed no changes, while others have found signs of dysfunction following strenuous, intense, and prolonged exercise [6,7,8,9,10,11,12]. Even less information is available on the role of efficient cardiac adaptations to exercise in the response of diastolic function to increased external physical workload. Doppler ultrasound supplies precise and valuable estimates of early (E) and late (A) transmitral flow, a tissue Doppler measurement of mitral annular velocity (e’ and a’, correspondingly), and estimations of LV filling pressure (E/e’). Diastolic stress echocardiography has been introduced to detect exercise-induced changes in diastolic filling pressures by performing Doppler echocardiography during exercise [13]. It was supported that isometric handgrip stress (IHE) echocardiography could differentiate between normal and abnormal diastolic function [14]. It is considered an excellent diagnostic tool for total body exercise since it avoids the known limitations of the latter for the perfect evaluation of LV filling function [15].

Therefore, this research aimed to study the possible different responses to diastolic loading ultrasound with isometric handgrip exercise in two groups of middle-aged healthy men with different levels of physical activity, classified as either active or sedentary. Moreover, a secondary objective of the study was to investigate the relationship between the demographic, hemodynamic, and echocardiographic measurements, along with the degree of LV diastolic function.

## 2. Materials and Methods

The participants were selected after completing and evaluating translated international physical activity questionnaires (IPAQ, Athens, Greece), distributed to 60 middle-aged men aged 41–51 in medical offices, sports venues, and gyms [16]. Exclusion criteria were the existence of any known morbidity (cardiac, metabolic, or other diseases), the regular use of any medication, the presence of pathological findings in a resting electrocardiogram or echocardiographic study, and the achievement of moderate weekly physical activity performance. According to the calculated METs × min/week, subjects were divided into two equal groups, i.e., those reporting intense physical activity (>3000 METs × min/week), who were not athletes, and those with low levels of physical activity (<300 METs × min/week). With the above inclusion and exclusion criteria, 20 healthy men were finally selected who were in their fifth decade. All participants were informed about the study’s purpose and procedures, and all provided written informed consent.

All participants underwent an echocardiographic study when at rest and at the peak of an isometric handgrip exercise intervention. The handgrip test was selected as a simple, easily applicable method to help objectively assess the current state of diastolic cardiac function using stress echocardiography [14]. Isometric handgrip exercise represents effective diastolic stress for heart function, as is proved by the high rise in blood pressure (both systolic and diastolic) and the rate pressure product. Thus, a handgrip with more than 30–40% of the Tmax for a prolonged time period (more than 2–3 min) is unsustainable. In our study, the handgrip load for all participants was 35% of their Tmax figure for 3 min. 

All participants arrived at the Sports Medicine Laboratory of the Aristotle University of Thessaloniki in the morning, with the recommendation of refraining from coffee and tobacco consumption that day. First, height and weight were determined (a SECA 220 stadiometer and SECA 803 digital scale) to calculate the body mass index (BMI) and body surface area (BSA). Then, with a digital arm sphygmomanometer (Omron M7 Intelli IT HEM-7322T-E), the resting systolic blood pressure (SBP), diastolic blood pressure (DBP), mean arterial pressure (MAP), and heart rate (HR) were measured. Afterward, the maximal hand traction force (Tmax), measured in kilograms, was determined as follows: each participant exerted a maximal influence with each hand against a hydraulic dynamometer (AC3330 dynamometer, MSD, Tisselt, Belgium) in three consecutive attempts. Each attempt lasted 5–6 s and was separated by one minute from the next to avoid burnout. Thus, the dominant upper extremity was determined, as well as its maximum strength, as the average of the three attempts. Finally, the rate pressure product (RPP) was calculated by multiplying the HR by SBP when at rest and IHE.

Moreover, each subject underwent a 12-lead resting electrocardiogram (MAC 600 electrocardiograph, GE, Milwaukee, WI, USA) and a two-dimensional and Doppler echocardiographic study (VIVID S5 echocardiograph with a 3Sc 2.5 MHz head, GE Vingmed Ultrasound AS, Horten, Norway) at the resting and peak points of an isometric handgrip exercise intervention.

The echocardiographic examination was performed by a cardiologist who is certified in adult transthoracic echocardiography by the European Association for Cardiovascular Imaging. Subjects were arranged in the left lateral recumbent position, and ultrasound images were obtained from 3 consecutive cardiac cycles in both the parasternal long axis and the four-chamber apical axis, which were stored so that they would be available for study and analysis after the end of the test. From the apical axis of the four cavities, in addition to the classic volumetric measurements, Doppler measurements were taken to study the diastolic function (via diastolic flow measurements with pulsed Doppler, taken by placing the volume sample at the top of the mitral cusps, as well as by determining the maximum tissue velocity of the lateral wall of the left ventricle at the level of the mitral annulus). At peak isometric exercise, there is limited time to manage to assess early (E) and late (A) diastolic mitral valve inflow velocities, as well as early tissue Doppler diastolic velocities from both the lateral and the septal site, recorded in three consecutive cardiac cycles before the strength of the participant was decreased because of exhaustion. Single-site measurements can be used in the presence of globally normal or globally abnormal LV systolic function [14]. In our study, all participants were healthy individuals with globally normal LV systolic function, and the lateral site was preferred. After the resting echocardiographic study, having already determined the Tmax, the IHE intervention began as follows: each subject exerted continuous pressure for 3 min against the hydraulic dynamometer with the dominant upper extremity and at an intensity of 35% of the maximum Tmax. Blood pressure and heart rate were measured during the last minute (peak) of the isometric exercise. Additionally, all echocardiographic indices were reassessed at IHE.

Statistical analysis: the statistical program, IBM SPSS Statistics v26.0, was used for the processing, presentation, and statistical analysis of the data. For statistical analysis of the sample variables, the Kolmogorov-Smirnov statistical test was applied, using the Monte Carlo simulation method for 10,000 samples, to investigate whether the data followed the normal distribution. At the same time, the sample identified the most basic graphs and descriptive statistics. Student’s *t*-test was used to compare the measurements between the inactive and active participants of the study. Furthermore, a paired samples *t*-test was used to compare the means of the measures taken when at rest and at the peak of the isometric exercise test. In addition, simple and multiple binomial logistic regression, as well as simple and multiple linear regression analysis, were applied while controlling for all their conditions to create predictive models for participants’ physical activity level (inactive vs. active) and the change in the ratio of left ventricular early diastolic filling velocities to the lateral wall tissue velocity (ΔE/e’), respectively. The level of statistical significance for all statistical tests was preset at *p* ≤ 0.05.

## 3. Results

Based on the Kolmogorov–Smirnov statistical test, using the Monte Carlo simulation method (for 10,000 samples), all sample variables followed the normal distribution (*p* > 0.05). Thus, parametric statistics were used.

The principal baseline data of both groups are presented in Table 1.

Table 2 shows the mean values of SBP, DBP, MBP, and HR when at rest and during IHE. There was no statistically significant difference between the active and inactive participants in terms of mean SBP, DBP, MBP, and RPP when at rest and IHE. However, all variables increased significantly at peak IHE (*p* < 0.005 for all changes). The mean HR when at rest was lower by 18% (*p* < 0.05) in the active than in the inactive individuals. A marginally significant difference was confirmed at the peak IHE among the two groups (*p* = 0.059). In terms of the average double product of blood pressure × heart rate (RPP) when at rest and at the peak of the test, this did not differ significantly between the active and inactive participants.

The echocardiographic measurements are presented in Table 3. Active participants showed a significantly larger left ventricular end-diastolic diameter (LVEDd), end-diastolic volume (LVEDv), and mass index (LVMi), compared to inactive participants.

The average velocity of the early diastolic filling of the left ventricle (wave E) when at rest and during peak exercise did not differ between the active and inactive participants (*p* = 0.298 and *p* = 0.492). In addition, the mean velocity of the E-wave was not different between the resting state and peak IHE (*p* = 0.251). The mean velocity of the left ventricular late diastolic filling (wave A) when at rest and during peak exercise did not differ between the active and inactive participants (*p* = 0.901 and *p* = 0.825). No difference was found between resting values and IHE in the two groups. The mean deceleration time of the left ventricular early filling flow (dt) when at rest and during peak exercise did not differ between the active and inactive participants (*p* = 0.895 and *p* = 0.595). The average dt did not vary between the at-rest and peak exercise values (*p* = 0.116). The average value of the ratio of the velocities of E and A waves (E/A) when at rest and at the peak of exercise did not differ between the active and inactive participants (*p* = 0.313 and *p* = 0.969). No difference was found between at-rest values and IHE in both groups. The average maximal tissue velocity of the lateral wall of the left ventricle (TDI lat e’) when at rest and during peak exercise did not differ between the active and inactive participants (*p* = 0.759 and *p* = 0.084). In addition, the mean maximal tissue velocity of the lateral wall of the left ventricle did not vary between at-rest and peak exercise values (*p* = 0.251).

The mean value of left ventricular early diastolic filling velocity to early lateral wall tissue velocity (E/e’) was not different between the active and inactive individuals when at rest (*p* = 0.764). In addition, neither group showed any differences in E/e’ values between at-rest and peak exercise values. However, at the peak of the test, the active participants revealed a lower average value for the E/e’ ratio, compared to the inactive ones (*p* = 0.051). Finally, for the active participants, the mean value of the change in the ratio of left ventricular early diastolic filling velocities to lateral wall tissue velocity (ΔE/e’) was significantly smaller than in the inactive participants (*p* = 0.019).

Significantly positive linear correlations existed between the resting E/e’ ratio and the peak DBP (r = 0.7) and MBP (r = 0.6). There were also significantly positive linear correlations between the peak Ε/e’ ratio, BMI (r = 0.5), and BSA (r = 0.6). In addition, a significant positive linear correlation was shown between the ΔΕ/e’ and the BSA (r = 0.5). Moreover, there was a significantly positive, linear correlation between the peak E/e’ ratio and the end-diastolic thickness of the interventricular septum (r = 0.5). A significant positive linear correlation also appears between the ΔΕ/e’ and the left ventricular end-diastolic diameter (r = −0.4).

The binary logistic predictive model used the level of physical activity of the participants as a dependent variable and the change in the ratio of the velocities of the early diastolic filling of the left ventricle to the maximum tissue velocity of the lateral wall as an independent variable; this was expressed by the equation below, which predicted that for each 1-unit increase in the change in ΔE/e’ ratio at peak isometric handgrip exercise, the probability that the participant was an intensely active middle-aged man was expected to decrease by 98.6%, or else that the less active one was; the more active one was, a more significant increase was expected in the change in ΔE/e’ as a result of the diastolic loading of the heart.

log (Active/Inactive) = −4.284 × ΔE/e’

Finally, two simple linear statistical predictive models were also suggested that present clinical interest. The first model used ΔΕ/e’ as a dependent variable, with body surface area as an independent variable.

Based on Table 4, the resulting equation of the first simple linear model was the following:

ΔE/e’ = −2.518 + 1.324 × BSA.

The second simple linear statistical model used ΔE/e’ as the dependent variable and the end-diastolic left ventricular diameter as the independent variable:

Based on Table 5, the resulting equation of the first simple linear model is the following:

ΔE/e’ = 2.183 − 0.042 × LVEDd.

The two equations predicted that an increase in body surface area by 0.1 m^2^ was expected to increase by 0.1324 units ΔE/e’; also, an increase in left ventricular end-diastolic diameter by 1 mm was likely to decrease by 0.042 units ΔE/e’. The results predicted highlight the interactions between obesity, athletic cardiac remodeling, and cardiac response to acute diastolic stress.

## 4. Discussion

The study’s actual results suggest that efficient physical activity on the part of healthy middle-aged men positively influenced the lusitropic properties of the heart by preventing an increase in filling pressures as an inappropriate response to increased afterload. The Doppler diastolic ultrasound measurements show that the E/e’ ratio did not differ between the active and sedentary groups of men when at rest (6.1 for the active and 5.9 for the inactive). However, at the peak of the IHE, the E/e’ ratio was higher for the inactive participants (mean E/e’ = 7.5 for the inactive and mean E/e’ = 5.8 for the active, *p* = 0.051). Notably, the difference between the two groups was found to have more significance (*p* < 0.019) regarding Δ change of the above ratio E/e’ (ΔE/e’).

It is known that aerobic exercise training contributes to the reduction of body weight, reduces adipose tissue, decreases oxidative stress, increases tissue sensitivity to insulin, and improves the lipid profile, thus protecting cardiovascular health [17]. Our results showed that the BMI and BSA were significantly lower in the active participants compared to the inactive ones. It is already known that prolonged endurance exercise reduces HR, SBP, and DBP when at rest, which leads to a balanced enlargement of ventricular mass and dimensions, resulting in enhanced cardiac performance, a decrease in end-diastolic pressure, and an improvement in vascular function [18]. However, no differences were found regarding the values of SBP, DBP, and MBP between the two groups in our study except for the HR, which was found to be lower in active men. Active participants showed a significantly larger LVEDd, LVEDv LV mass, and left atrial volume index compared to inactive ones. The mean IVSd and LVEF values were similar in the two groups.

The relationship between physical activity, age-related cardiac remodeling, and diastolic function remains challenging. It has been reported that regular exercise improves LV compliance and myocardial tissue relaxation, and also reverses diastolic dysfunction in some pathologic conditions [4,19]. The mechanism by which physical activity was inversely related to the prevalence of impaired LV relaxation has yet to be identified. It has been suggested that endurance training maintains or increases vascular elasticity and, thus, produces a smaller arterial load [20]. It enhances LV function by decreasing the collagen content, reducing preventing fibrosis, increasing angiogenesis, decreasing mitochondrial dysfunction, and preventing cardiomyocyte apoptosis with aging [20,21,22]. Indeed, physical activity reduces oxidative stress and abdominal fat, which have been proposed as additional factors that impair myocardial relaxation [23]. Using transmitral Doppler analysis, d’Andrea et al. showed that there was a higher Em and Em/Am ratio, as well as a longer relaxation time in top-level endurance athletes, both at the septal and at the inferior wall levels, with comparable Sm and pre-contraction and contraction times, in comparison with strength-training individuals [8]. Caselli et al. found that Olympic athletes had similar E velocities but showed significantly decreased A velocities compared to controls, with increased E/A ratios [10]. In addition, isovolumic relaxation and deceleration times were longer in athletes than in controls. The E/e’ ratio was also slightly higher in athletes. Conversely, our study results showed that there were no differences regarding all LV diastolic indices between the active and not active men when at rest. The above discrepancies may be explained by the fact that our subjects were active men but not elite athletes. Similarly, Gates et al. reported that habitual aerobic endurance exercise status was not uniformly associated with the favorable modulation of age-associated changes in LV structure and diastolic function in men [24]. Moreover, a higher level of daily physical activity was found to have a limited effect on age-related changes in concentric remodeling, diastolic function, and cardiac performance in women [25]. Conversely, Cicec et al. reported that aerobic exercise effectively improved the diastolic function of the heart in sedentary women [26]. Tude Rodrigues et al. also found a significant increase in tissue Doppler Sm and Em velocities at the septal and lateral walls after six months of moderate-intensity aerobic training by sedentary men. In contrast, the Am velocities were unchanged. Tissue Doppler Em or Am velocities were not correlated with other echocardiographic or exercise variables [27].

Although the physiology of LV diastolic function is complex, the main mechanisms contributing to the cardiac filling phase can be differentiated into intrinsic and extrinsic. Preliminary results showed that stress echocardiography is technically feasible for demonstrating the change in E/e’ and mainly the change in this ratio (ΔE/e’) at the peak of isometric handgrip exercise, i.e., at the peak of the intense diastolic loading that occurs with exercise [15,28]. Therefore, this novel technique can noninvasively demonstrate the hemodynamic consequences of an exercise-induced increase in LV filling pressure [14,15,29]. The reflex begins with the contraction of the involved small muscle groups of the hand (IHE). This stimulates receptors that are sensitive to either mechanical strain or the metabolic products of exercise. Then, it provides stimuli to the cardiovascular system through efferent sympathetic pathways, resulting in increased heart rate and blood pressure. To smoothly pump blood during the systolic phase in this increased afterload condition, a significant increase in intracellular calcium is observed to maximize cardiac contraction [30]. However, this excess calcium must either be reabsorbed by the sarcoplasmic reticulum, to be reused in the next cardiac cycle, or must be expelled from the myocyte during the expansion phase. Any possible dysfunction in the above process will result in the prolonged connection of actin-myosin bridges, the disruption of ventricular dysfunction, and, ultimately, increased rigidity and increased filling pressure of the left ventricle [31]. Therefore, IHE has the potential to distinguish between normal and pathological cardiac conditions [32]. Hamatani et al. demonstrated an increase in end-diastolic and end-wedge pressure in patients with exertional dyspnea and functional mitral valve insufficiency [33]. Garg et al. observed a pathological increase in blood pressure with the isometric handgrip exercise in the healthy young adult offspring of hypertensive parents [34]. Manolas studied the pathological diastolic processes in hypertensive patients and patients with coronary artery disease and induced myocardial ischemia in response to isometric handgrip exercise [35]. Finally, Samuel et al. established IHE as, at least, an equal diastolic loading technique to the more commonly used and most extensively studied cycle ergometer; IHE avoids the respiratory and movement artifacts presented in the cycle ergometer test. Moreover, they demonstrated its utility in the differential diagnosis of the cardiac etiology of exertional dyspnea from extracardiac causes and when studying the diastolic cardiac response across the spectrum of heart failure syndrome [14,15].

Taking into account these conflicting views regarding the LV diastolic function of active individuals when at rest, we compared the effects of IHE in these individuals with those in the corresponding sedentary ones. The mean deceleration time of LV early-filling flow (dt) at rest and during peak exercise did not differ between the active and inactive participants, nor between the rest and peak exercise groups in our study. In addition, the E/A ratio did not differ between the two groups at rest and at the peak of exercise, and between at-rest and peak effort values. However, although no significant difference was observed in the two groups regarding the E/e’ ratio when at rest, this variable increased markedly at the maximum of the IHE in the sedentary subjects. Even more significant was the increase in ΔE/e’. More specifically, the active men responded to the diastolic stress stimulus by reducing ΔE/e’, compared to the one they had when at rest (the ΔE/e’ was −0.023). Conversely, the inactive individuals responded to loading by increasing the ΔE/e’ compared to the corresponding value when at rest (ΔE/e’ was 0.31). Similarly, Samuel et al. reported that diastolic function in young, healthy individuals was well preserved in response to IHE, establishing normal cardiac function [14]. Moreover, the researchers used that method to distinguish between a normal and abnormal diastolic stress response in a group of seniors with age-related resting diastolic impairments and in clinically stable, well-characterized HFpEF patients with severe exercise intolerance [14,15]. The authors attributed this different response to impaired intracellular calcium-handling in the two groups. Specifically, transient increases in intracellular calcium values in a weakened myocardium led to prolonged actin-myosin cross-bridge formation and increased myocardial stiffness. Conversely, in a study of middle-aged healthy subjects, Ha et al. reported that mitral inflow (E and A) and annular velocities (E’ and A′) increased significantly; still, the E/A and E/E’ ratios did not change significantly following a symptom-limited treadmill exercise test [36].

Our study showed significant positive linear correlations between the resting E/e’ ratio and the peak DBP and MBP. Other studies have demonstrated a significant correlation between the LV afterload-associated variables (SBP, DBP, MBP) and changes in the global myocardial longitudinal strain and myocardial work indices [37,38]. Furthermore, the present study showed significant positive linear correlations between the peak Ε/e’ ratio, BMI, and BSA. In addition, a significant positive linear correlation was found between the ΔΕ/e’ and the BSA. Given that regular exercise training benefits the physique, these correlations are attributed to the above finding. Moreover, our study showed a significant positive, linear correlation between the peak E/e’ ratio and the end-diastolic thickness of the interventricular septum. A significant positive linear correlation was also observed between the ΔΕ/e’ and the left ventricular end-diastolic diameter. Furthermore, D’Andrea et al., in multivariate analyses, evidenced an independent positive association between Em peak velocity and LV end-diastolic volume, and an independent correlation of global longitudinal strain with the sum of LV wall thicknesses. In addition, the degree of LV remodeling (i.e., LV end-diastolic volume and mass) and the left atrium size have been found to be crucial well-defined determinants of the E/e’ ratio [39]. Finally, LA volume was strongly associated with diastolic function grade, independent of LV ejection fraction, age, gender, and cardiovascular risk score [40].

The small number of participants is one limitation of the current study. This fact significantly limits the interpretation of the regression analysis results and the predictive models. Another weakness was the fact that the functional capacity of the individuals was assessed based on the IPAQ questionnaire and not by maximum cardiopulmonary exercise testing. In addition, only males participated in the study. Finally, the results of the diastolic indices between groups were not statistically significant and only a marginally statistically significant difference was found regarding the delta change of the E/e’ ratio. The above limitations do not allow us to support our study’s clinical significance safely. However, from statistical logistic predictive models, we suggested interactions between obesity, cardiac adaptations to exercise training, and cardiac response to acute diastolic stress. On the contrary, some authors limit the usefulness of isolated indices of diastolic function. Thus, it was suggested that using E/e’ to estimate LV filling pressures is controversial, failing to correlate the values with invasive measurements in various clinical situations [41]. Mitter et al. also verified that E/A and E/e’ should not be used alone to assess diastolic function in clinical practice but, rather, in combination with clinical data such as age and medical history, as well as other critical echocardiographic parameters for the accurate assessment of LV diastolic relaxation [42].

In conclusion, the study’s results demonstrated that active middle-aged men respond perfectly to the intense diastolic stress caused by isometric handgrip exercise without a significant change in ΔE/e’ and, therefore, without evidence of an increase in left ventricular end-diastolic pressure, improving the lusitropic properties of the heart. In contrast, the significant difference in the ΔE/e’ ratio observed in the inactive middle-aged men indicates an increase in LV end-diastolic pressures as an attempt to overcome the increased afterload of isometric handgrip exercise.

## Figures and Tables

**Table 1:** Baseline data between active and inactive groups of men.

Variables	Active Group	Inactive Group	*p*
Μean age (years)	45.6 ± 2.55	45.1 ± 1.0	0.591
BMI (kg/m^2^)	24.72 ± 2.09	27.93 ± 2.88	0.05
BSA (m^2^)	1.93 ± 0.09	2.09 ± 0.10	0.05
METs × min/week	3937 ± 3268	284 ± 181	0.003
max IHE (kg)	38.14 ± 4.72	40.25 ± 5.93	0.390

Abbreviations: BMI, body mass index; BSA, body surface area; IHE, isometric handgrip exercise.

**Table 2:** Hemodynamic data between the active and inactive groups of men when at rest and in response to IHE.

Variables Hemodynamics	Active Group	Inactive Group	*p*
SBP, REST, (mmHg)	125.7 ± 17.6	122.5 ± 10.9	0.631
SBP, IHE, (mmHg)	166.8 ± 11.0	162.6 ± 12.8	0.442
DBP, REST, (mmHg)	71.3 ± 11.5	74.0 ± 11.0	0.598
DBP, IHE, (mmHg)	107.2 ± 9.7	103.0 ± 11.4	0.387
MAP, REST, (mmHg)	89.4 ± 12.4	90.2 ± 10.5	0.878
MAP, IHE, (mmHg)	127.0 ± 8.7	122.9 ± 11.2	0.371
HR, REST, (bpm)	61.4 ± 11.4	74.8 ± 9.1	0.01
HR, IHE, (bpm)	75.6 ± 10.9	83.9 ± 7.2	0.059
RPP, REST, (mmHg × bpm)	7783 ± 2382	9222 ± 1796	0.144
RPP, IHE, (mmHg × bpm)	12,654 ± 2329	13,664 ± 1790	0.291

Abbreviations: SBP, systolic blood pressure; DBP, diastolic blood pressure, MAP, mean arterial pressure; HR, heart rate; RPP, rate pressure product; IHE, isometric handgrip exercise.

**Table 3:** Echocardiographic data, comparing active and inactive men when at rest and during IHE.

Variables	Active Group	Inactive Group	*p*
LVEDd (mm)	50.4 ± 3.1	46.2 ± 2.4	0.003
IVSd (mm)	10.2 ± 1.3	10.3 ± 1.4	0.872
PWd (mm)	9.5 ± 1.18	9.4 ± 0.97	0.838
LV mass (gr)	208.40 ± 34.12	186.8 ± 34.34	0.175
LV mass index (gr/m^2^)	107.70 ± 16.03	89.2 ± 13.32	0.012
LVEF (%)	65.4 ± 4.0	62.2 ± 3.9	0.089
SV, REST, (mL)	86.7 ± 14.9	79.6 ± 17.4	0.340
LVEDV, (mL)	117.50 ± 20.07	99.40 ± 12.78	0.027
LAVi, mL/m^2^	29.7 ± 6.7	22.6 ± 4.0	0.010
**LV Doppler**			
MV Ε velocity, REST, (m/s)	0.704 ± 0.11	0.65 ± 0.12	0.298
MV E velocity IHE, (m/s)	0.69 ± 0.16	0.74 ± 0.15	0.492
MV A velocity, REST, (m/s)	0.54 ± 0.13	0.54 ± 0.07	0.901
MV A velocity, IHE, (m/s)	0.66 ± 0.13	0.67 ± 0.10	0.825
E/A ratio, REST	1.39 ± 0.45	1.22 ± 0.23	0.313
E/A ratio, IHE	1.1 ± 0.37	1.09 ± 0.17	0.969
MV deceleration time, REST, (m/s)	200.40 ± 18.81	201.70 ± 24.40	0.895
MV deceleration time, IHE, (m/s)	193.10 ± 20.57	188.00 ± 21.59	0.595
LV lateral e’ velocity, REST, (m/s)	0.12 ± 0.03	0.12 ± 0,03	0.759
LV lateral e’ velocity, IHE, (m/s)	0.12 ± 0.03	0.10 ± 0.02	0.084
LV E/e’ ratio, REST	6.07 ± 1.17	5.87 ± 1.76	0.764
LV E/e’ ratio, IHE	5.78 ± 1.27	7.50 ± 2.27	0.051
LV ΔE/e’	−0.02 ± 0.26	0.31 ± 0.32	0.019

Abbreviations: LVEDd, left ventricular end-diastolic diameter; IVSd, interventricular septum end-diastolic thickness; PWd, posterior wall end-diastolic thickness; LVEF, left ventricular ejection fraction; SV, stroke volume; LVEDV, left ventricular end-diastolic volume; IHE, isometric handgrip exercise; LAVI, left atrial volume index; MV, mitral valve; LV, left ventricle.

**Table 4:** Model regression coefficients and the results of the *t*-tests regarding BSA.

Regression Coefficients	Coefficient b	*t*-Test	*p*
Fixed term	−2.518	−2.251	0.037 *
BSA	1.324	2.384	0.028 *

* There is a statistically significant correlation between the two variables (*p* < 0.05).

**Table 5:** Model regression coefficients and the results of the *t*-tests regarding LVEDd.

Regression Coefficients	Coefficient b	*t*-Test	*p*
Fixed term	2.183	2.230	0.039 *
LVEDd	−0.042	−2.088	0.051 *

* There is a statistically significant correlation between the two variables (*p* < 0.05).

## Data Availability

Data are available on personal request to the corresponding author.
